# Investigation of In Situ Gelation Behavior and Enhanced Oil Recovery Ability of Polymer Gel Used for Controlling CO_2_ Channeling in Tight Fractured Reservoir

**DOI:** 10.3390/gels10110741

**Published:** 2024-11-14

**Authors:** Hong He, Yibo Liu, Guang Zhao, Yifei Liu, Haihua Pei, Wei Zhou

**Affiliations:** 1 College of Petroleum Engineering, Yangtze University, Wuhan 430100, China; hehong1103@163.com; 2 Hubei Key Laboratory of Oil and Gas Drilling and Production Engineering, Yangtze University, Wuhan 430100, China; 3 Shandong Key Laboratory of Oilfield Chemistry, China University of Petroleum (East China), Qingdao 266580, China; zhaoguang@upc.edu.cn (G.Z.); liuyifei@upc.edu.cn (Y.L.); peihaihua@upc.edu.cn (H.P.); 4 College of Petroleum Engineering, China University of Petroleum (East China), Qingdao 266550, China; 5 College of Earth Science and Resources, Chang’an University, Xi’an 710100, China; zhouwei1666@126.com

**Keywords:** gelation behavior, polymer gel, CO_2_ channeling, enhanced oil recovery, tight fractured reservoir

## Abstract

Polymer gels are one of the most common plugging agents used for controlling CO_2_ channeling and improving sweep efficiency and oil recovery in tight fractured reservoirs. However, the in situ gelation behavior and enhanced oil recovery ability of polymer gel in fractured porous media is still unclear. Thus, in this study, the bulk and in situ gelation behavior of crosslinked phenolic resin gel in a long stainless microtube as the fractured porous media was investigated. The enhanced oil recovery ability of phenolic resin gel used for CO_2_ channeling was investigated by means of a fractured core model. Results show that, with the increase of polymer and crosslinker concentrations, the bulk gelation time shortens and gel strength improves during the static gelation process. With the increase of polymer concentration and temperature, the in situ static gelation time and dynamic gelation time of the gel system in the microtube are shortened, and the breakthrough pressure gradient increases after gelation. Compared with the in situ static gelation behavior, the in situ dynamic gelation time is prolonged and the breakthrough pressure gradient decreases after gelation. The in situ static gelation time in the microtube is 1.2 times that of bulk gelation time in an ampoule bottle, and the in situ dynamic gelation time is nearly 3 times that of ampoule bottles. When the injected slug volume was 1.0 FV (fracture volume), as the polymer concentration increased from 3000 mg·L^−1^ to 4000 mg·L^−1^, the incremental oil recovery increased from 3.53% to 4.73%.

## 1. Introduction

As conventional oil resources are depleted, it is imperative to develop unconventional oil resources [1,2]. For conventional reservoirs, when the reservoir is depleted after primary production, water flooding is one of the most widely used techniques for enhanced oil recovery by supplementing reservoir energy and displacing remaining oil [3]. However, due to the permeability contrast between the matrix and existence of induced fracture networks, it is inevitable that a large percentage of oil remains unrecovered in the matrix after water flooding [4,5,6,7]. In terms of enhanced oil recovery in tight oil reservoirs, there have been studies focused on CO_2_ injection for tight oil exploration [8,9,10,11,12,13], but due to the existence of fractures, gas channels through the fracture and gas breakthrough occurs, which can lead to low sweep efficiency and early breakthrough. To alleviate these problems, different methods have been adopted to control gas channeling, including water-alternating-gas (WAG) and a chemical plugging technique. The CO_2_ water-alternating-gas (CO_2_-WAG) technique has been proposed and successfully applied to control gas mobility and achieve higher recovery efficiency [14,15,16,17]. However, for a very heterogeneous reservoir, the WAG cannot achieve good results. Therefore, the chemical plugging technique has usually been applied to control gas channeling in a fractured reservoir. In a previous study, different plugging agents including CO_2_ thickening agent, foam and polymer gel have been extensively investigated [18,19]. Many different kinds of CO_2_ thickening agent have been tested, including fluoropolymers, siloxane polymers, tailor-made surfactants and small molecule compounds that can be directly dissolved in supercritical CO_2_ as chemical additives to increase the viscosity of supercritical CO_2_ under a reservoir condition. In addition, in a previous study, a CO_2_-responsive gel and a CO_2_-responsive preformed particle gel have also been reported. Dai et al. prepared a kind of CO_2_-reponsive gel composed of small molecular amine compounds and a modified long-chain alkyl anionic surfactant used for gas channeling plugging. Deng et al. developed types of CO_2_-responsive preformed particle gels (CR-PPGs) with high strength to address CO_2_ gas channeling problems during CO_2_ flooding in fractured reservoirs [20,21]. Although many kinds of CO_2_ thickening agent have been developed [21,22], CO_2_ thickening agents with oilfield popularization value are still subject to research.

Foam can effectively reduce the relative permeability of gas in porous media, thus effectively controlling gas mobility during the process of CO_2_ injection [23]. Foam can be formed by a mixing foaming agent with different gas types, including air, nitrogen and CO_2_ under external force. Due to the advantages of high apparent viscosity and selective plugging ability, foam has been widely applied in mature water flooding reservoirs. However, for high temperature and high salinity reservoirs, foaming ability and foam stability are not satisfactory, which can affect the abilities of sweep efficiency expanding and oil recovery. To improve foam stability, polymer reinforced foam, gel foam and three-phase foam were developed [24]. These foams has been successfully applied in mature high permeability sandstone reservoirs; however, foam plugging agent has application limitations for fractured reservoirs.

Polymer gels are one of the most common plugging agents used for controlling gas channeling and improving sweep efficiency [25,26,27]. Polymer gels are formed by polymer and crosslinkers including chromium gel and phenolic resin gel. Due to their short gelation time and bad thermal stability, organically crosslinked gels such as phenol–formaldehyde polymer gel and phenolic resin gel were used. Polymer gel treatment has been recognized as a cost-effective method to control water production in mature reservoirs. Some remarkable achievements have been made in the field application [28,29], but some problems still exist during gel treatment in the field. The in situ gelation behavior in porous media is especially related to the success rate of gel treatment [30,31,32,33,34]. Usually, before the field gel treatment, bulk gelation behavior in ampoule bottles is evaluated by the bottle test method under static conditions to determine the gelation time, which can provide a basis for the gel placement. However, during the injection process and shut-in period of the gel treatment, the in situ gelation behavior in porous media is different from bulk gelation behavior. The in situ gelation behavior, including static gelation and dynamic gelation in porous media, is often evaluated by conducting core flow experiments. The difference between bulk gelation and in situ gelation behavior evaluation is as follows: (1) The gelation circumstances are different. For bulk gelation behavior evaluation, the bulk gelation environment is an ampoule bottle, while for in situ gelation behavior evaluation, the in situ gelation environment is porous media. (2) There is a difference between static gelation and dynamic gelation. Bulk gelation behavior in an ampoule bottle is evaluated under static conditions. However, for in situ gelation behavior, during the gelant solution flow in porous media, due to the dilution, shear, adsorption and retention effects from porous media, the in situ dynamic gelation behavior in porous media is different.

In previous studies, the bulk and in situ gelation behavior of gelant solutions in consolidated porous media has been extensively investigated [35,36,37,38]. It was demonstrated that the bulk gelation behavior was different from in situ gelation behavior in aspects such as gelation time and gel strength. However, to the best of our knowledge, little research has been done on in situ gelation behavior in fractured porous media, which is crucial for the design of the injection amount and injection rate of polymer gel. It is difficult to provide a basis for the design of the gelant formulation and injection parameters during field tests. Therefore, it is necessary to evaluate some gelation behavior issues as follows: (1) The differences in gelation time and gel strength between bulk and in situ gelation in fractured porous media. (2) The influence of injection rate on in situ dynamic gelation behavior in fractured porous media, which affects the channeling effect of gel in tight fractured reservoirs.

Moreover, the plugging ability of polymer gel in fractured reservoirs can affect the enhanced oil recovery ability. In a previous study, the parallel sandpack mode or short core model was used to evaluate the enhanced oil recovery of polymer gel, which cannot actually reflect the enhanced oil recovery ability for tight fractured reservoirs. Thus, it is crucial to design a proper fractured core model to evaluate the enhanced oil recovery ability of polymer gel used for controlling CO_2_ channeling.

Therefore, the objective of this study is as follows: (1) A long stainless slim tube was used as the fractured porous media to investigate bulk gelation behavior and in situ gelation behavior during flowing, which can contribute to understanding the differences between bulk gelation and in situ gelation in fractured porous media. The bulk gelation behavior testing was focused on the gelation behavior of polymer gel in an ampoule bottle by the bottle method. The in situ gelation behavior included static gelation and dynamic gelation behavior. (2) Based on the findings in relation to gelation behavior, a fractured core was used to investigate the enhanced oil recovery ability of phenolic resin gel. This study can ensure the plugging effect of polymer gel used for fractured low permeability tight reservoirs and provide a theoretical basis for the design of the construction parameters of the polymer gel.

## 2. Results and Discussion

### 2.1. Bulk Gelation Behavior

#### 2.1.1. Effect of Polymer and Crosslinker Concentration

**Figure 1:**
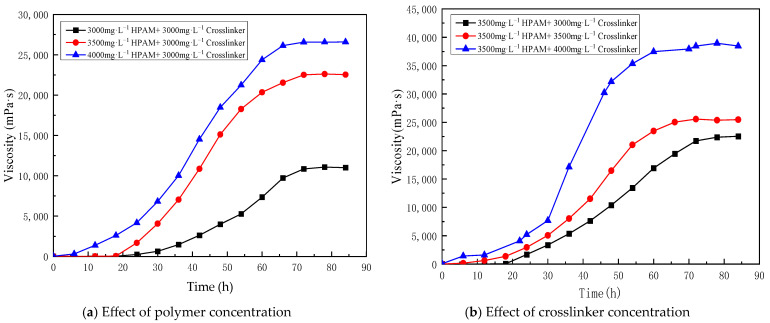
The effect of polymer and crosslinker concentration on the bulk gelation process at 70 °C.

The influences of the polymer concentration, crosslinker concentration and polymer/crosslinker concentration ratio on bulk gelation behavior of phenolic resin gel were investigated by conducting a series of bottle test experiments at 70 °C. The influences of the polymer concentration and polymer/crosslinker concentration ratio were studied by varying the polymer concentration, whereas the crosslinker concentration remained constant. Similarly, the influence of the crosslinker concentration was studied by varying the crosslinker concentration, whereas the polymer concentration remained constant. The experimental results of Effect of Polymer and Crosslinker Concentration are shown in Figure 1.

It can be obviously noted that, with the prolongation of time, the viscosity of the gelant solution changed slightly, and then increased rapidly until it became stable. The bulk gelation process can be divided into three stages: induction stage, gelation stage and stable stage. When the viscosity of the gelant solution begins to rise significantly, the gelation time is defined as the time corresponding to the inflection point between the induction stage and the gelation stage. According to the above figures, the gelation time and gel strength of polymer gel to be formed under different polymer and crosslinker concentration components are obtained, as shown in Table 1.

The gelation time and strength are adjustable at 70 °C; when the concentration of polymer and crosslinker is in the range of 3000 mg·L^−1^ to 4000 mg·L^−1^, the gelation time ranges from 17.5 h to 40 h, and the gelation strength ranges from 11,006 mPa·s to 38,457 mPa·s. With the increase of polymer and crosslinker concentrations, the gelation time of the gelant solution is shortened and the gelation strength increases. When the concentrations of polymer and crosslinker increase, the number of active points participating in the crosslinking reaction increases, which improves the contact reaction probability of reaction groups, speeds up the crosslinking reaction rate, shortens the gelation time and increases the gelation strength.

#### 2.1.2. Effect of Temperature

**Figure 2:**
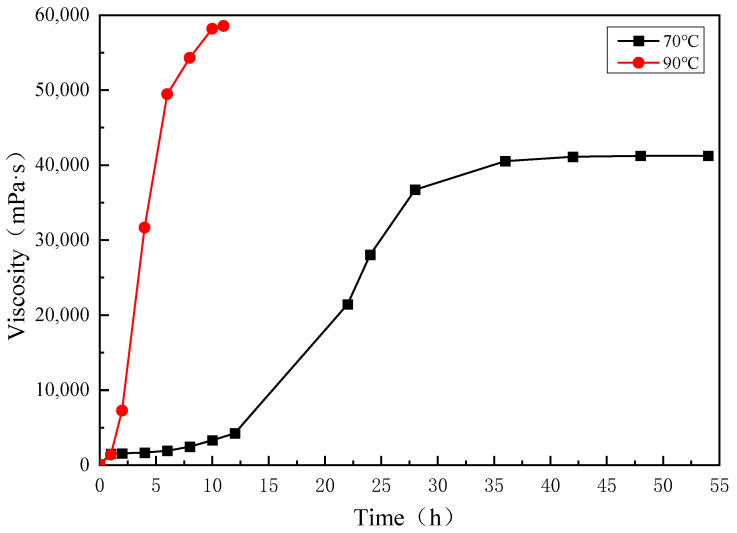
The effect of temperature on bulk gelation behavior (70 °C and 90 °C).

**Figure 3:**
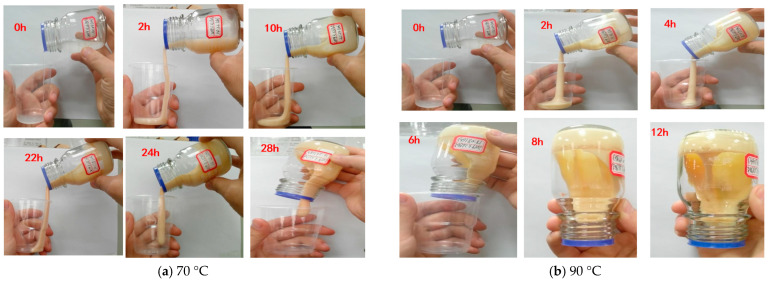
Comparisons of gel appearance varying with time at 70 °C and 90 °C.

A long stainless steel microtube (Φ 1.0 mm × 100 mm) physical simulation model was established and continuous injection of gelant solution was used to investigate the in situ dynamic gelation behavior during flow in the long microtube. When the gelant solution system flows out of the microtube, this will lead to the failure of the dynamic gelation experiment. Due to the limitation of long gelation time at 70 °C and the length of microtube model, in order to better analyze and compare the gelation behavior under bulk and in situ gelation conditions, the gelation property of a gelant solution composed of 4000 mg·L^−1^ polymer and 4000 mg·L^−1^ crosslinker was studied at 90 °C. Figure 2 shows the viscosity of the gelant solution varying with time under different temperatures, at 70 °C and 90 °C. Figure 3 shows the appearance of the gelant solution varying with time under different temperatures of 70 °C and 90 °C.

The experimental results are shown in Table 2. When the concentration of polymer and crosslinker is constant (4000 mg·L^−1^ polymer + 4000 mg·L^−1^ crosslinker), gelation time decreases from 10 h at 70 °C to 2 h at 90 °C. The gelation strength increases from 41,245 mPa·s at 70 °C to 58,553 mPa·s at 90 °C. With the increase of temperature, the molecular movement of the polymer and crosslinker accelerates, and the crosslinking reaction probability of the reaction groups increases, which increases the crosslinking reaction rate, shortens the gelation time and increases the gel strength.

### 2.2. In Situ Static Gelation Behavior

#### 2.2.1. Effect of Temperature on In-Situ Static Gelation

**Figure 4:**
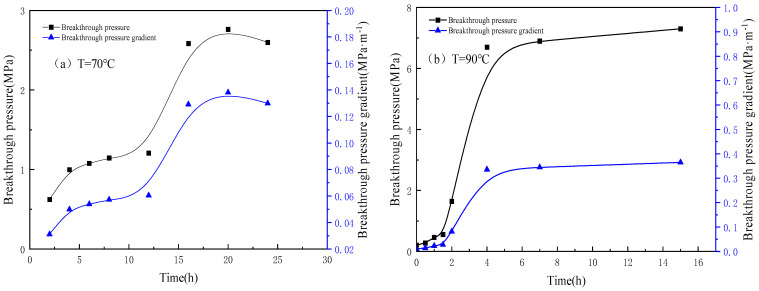
Breakthrough pressure and pressure gradient versus time at 70 °C and 90 °C.

The in situ static gelation behavior of phenolic resin gel was studied by conducting slim tube flow experiments. A stainless microtube (Φ 1.0 mm × 20 m) was used to simulate a single fracture in porous media. According to the experimental procedures of the in situ static gelation behavior test, the breakthrough pressure and pressure gradient versus time at 70 °C and 90 °C were as shown in Figure 4. Moreover, the water breakthrough pressure gradient after in situ static gelation in the microtube was measured, as shown in Table 3.

As shown in Figure 4, under temperatures of 70 °C and 90 °C, with the lengthening of the standing time of the gelant solution system in the microtube, the breakthrough pressure gradient of the gel system increased, slowly at first, and then increased rapidly until it was basically stable. The in situ static gelation process of the gel system in the microtube is divided into induction, gelation and stability stages. The time corresponding to the inflection point between the induction stage and the gelation stage, when the breakthrough pressure gradient begins to rise significantly, is defined as the gelation time. Table 3 shows the in situ static gelation time and gel strength at 70 °C and 90 °C. For the gel system composed of 4000 mg·L^−1^ polymer and 4000 mg·L^−1^ crosslinker, at 70 °C, the in situ static gelation time in the microtube is 12 h, and the breakthrough pressure gradient is 0.138 MPa·m^−1^. At 90 °C, the in situ static gelation time of the gel system in the microtube is 1.5 h, and the breakthrough pressure gradient is 0.365 MPa·m^−1^. As the temperature increases, the in situ static gelation time shortens, and the subsequent water flooding breakthrough pressure gradient increases, reflecting the increase in the gel strength. The reason for this is that with the increase of temperature, the movement of polymer and crosslinking agent molecules accelerates, the probability of a collision contact crosslinking reaction between reactive groups increases, the crosslinking reaction speed accelerates, the gelation time shortens, and the gel strength increases.

#### 2.2.2. Effect of Concentration on In-Situ Static Gelation

**Figure 5:**
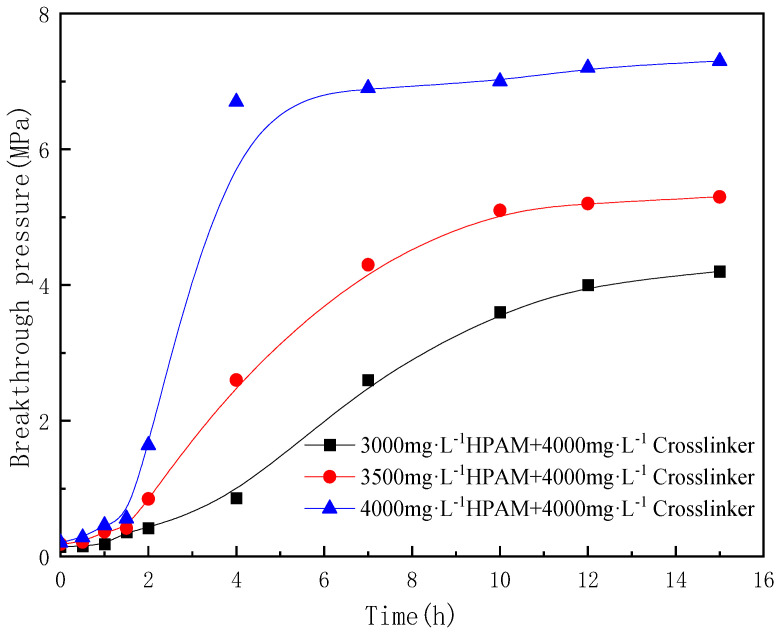
Breakthrough pressure and pressure gradient of different polymer concentrations versus time at 90 °C.

The influence of the polymer concentration was studied by varying the polymer concentration, whereas the crosslinker concentration remained constant. Figure 5 shows the water breakthrough pressure gradient versus time at 90 °C. Moreover, the water breakthrough pressure gradient after in situ static gelation in microtube was also measured, as shown in Table 4.

When the crosslinker concentration is 4000 mg·L^−1^, as the polymer concentration increases from 3000 mg·L^−1^ to 4000 mg·L^−1^, the in situ static gelation time in the microtube decreases from 3.5 h to 1.5 h, and the breakthrough pressure gradient increases from 0.21 MPa·m^−1^ to 0.365 MPa·m^−1^. With the increase of polymer concentration, the in situ static gelation time in the microtube shortens, and the subsequent water flooding breakthrough pressure gradient increases. The reason for this is that the number of amide groups participating in the crosslinking reaction increases, thereby increasing the probability of a contact reaction between reaction groups, accelerating the crosslinking reaction rate, shortening the gelation time, and increasing the gelation strength.

### 2.3. In-Situ Dynamic Gelation Behavior

A long stainless steel microtube (φ 1.0 mm × 100 m) physical simulation model was established and continuous injection of gelant solution was used to investigate the in situ dynamic gelation behavior during flow in the long microtube. When the gelant solution system flows out of the microtube, it will lead to the failure of dynamic gelation experiment. Due to the limitation of long gelation time at 70 °C and the length of the microtube model, in order to better analyze and compare the gelation behavior under bulk and in situ gelation conditions, the effects of temperature and polymer concentration on the gelation properties of gelant solution were studied.

#### 2.3.1. Effect of Temperature on Dynamic Gelation Behavior

**Figure 6:**
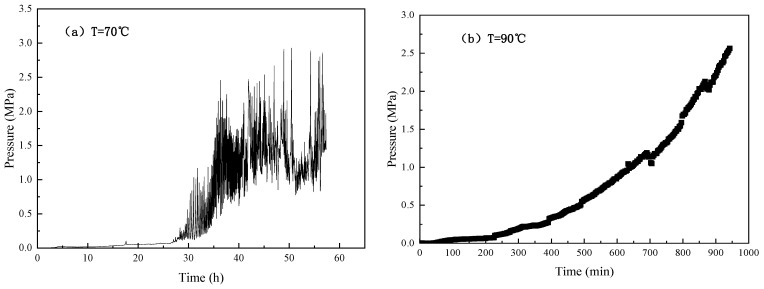
Change of injection pressure with time at 70 °C and 90 °C (injection rate v = 0.05 mL·min^−1^).

Due to the limitation of long gelation time at 70 °C and the length of the microtube model, to compare the gelation behavior under bulk and in situ gelation conditions, the effect of temperature on the in situ dynamic gelation property of the gelant solution was studied. Figure 6 shows the change of injection pressure versus time at 70 °C and 90 °C. After 1.0 PV gelant solution was injected, subsequent water flooding was carried out to determine the breakthrough pressure. The breakthrough pressure is defined as the maximum injection pressure, that is, the strength of gel in microtube after dynamic gelation is determined. Table 5 summarizes the in situ dynamic gelation time and gel strength of gelant solution under different temperatures.

According to the gelation time determination method, as the temperature increases from 70 °C to 90 °C, the in situ dynamic gelation time decreases from 32.5 h to 4.5 h and the gel strength increases from 0.0325 MPa·m^−1^ to 0.04 MPa·m^−1^. As the temperature increases, the in situ dynamic gelation time shortens, and the in situ dynamic gel strength increases.

#### 2.3.2. Effect of Concentration on Dynamic Gelation Behavior

**Figure 7:**
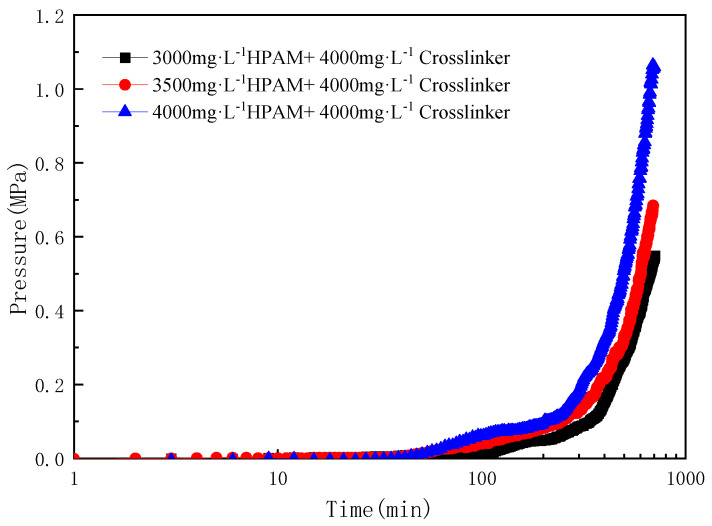
Change of injection pressure as a function of time during flow of gelant solution in the microtube (90 °C, 0.25 mL·min^−1^).

The influence of polymer concentration on in situ dynamic gelation behavior of phenolic resin gel was investigated by conducting a series of flow experiments at 90 °C. The polymer concentration was 3000 mg·L^−1^ The crosslinker concentration was in the range of 3000 mg·L^−1^ to 4000 mg·L^−1^. The gelant solution was injected into the microtube continuously at an injection rate of 0.25 mL·min^−1^. Figure 7 shows the injection pressure versus time during flow of gelant solution in the microtube.

After 1.0 PV gelant solution was injected, subsequent water flooding was carried out to determine the breakthrough pressure. The breakthrough pressure is defined as the maximum injection pressure, that is, the strength of gel in the microtube after dynamic gelation is determined. Table 6 summarizes the in situ dynamic gelation time and gel strength of gelant solution under different injection rates.

At 90 °C, when the crosslinker concentration is 4000 mg·L^−1^ and the polymer concentration ranges from 3000 mg·L^−1^ to 4000 mg·L^−1^, the in situ dynamic gelation time ranges from 3.9 h to 5.8 h and the gelation strength ranges from 0.0132 MPa·m^−1^ to 0.026 MPa·m^−1^. With the increase of polymer concentration, the gelation time decreases and gel strength increases. The number of active reaction groups participating in the crosslinking reaction increases, which improves the crosslinking reaction probability of the reaction groups, increases the crosslinking reaction rate, shortens the gelation time and increases the gelation strength.

#### 2.3.3. Effect of Injection Rate

**Figure 8:**
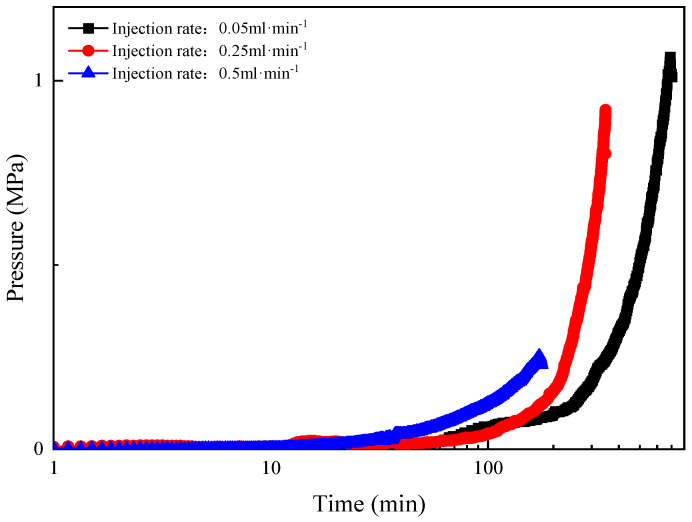
Change of injection pressure as a function of time during flow of gelant solution in the microtube at different injection rates (90 °C).

Due to the limitation of long gelation time at 70 °C and the length of the microtube model, the influence of injection rate on in situ dynamic gelation behavior of phenolic resin gel was investigated by conducting a series of in situ dynamic flow experiments at 90 °C. The concentrations of polymer and crosslinker were 4000 mg·L^−1^. The gelant solution was injected into the microtube continuously at different injection rates, including 0.05 mL·min^−1^, 0.25 mL·min^−1^ and 0.5 mL·min^−1^. Figure 8 shows the injection pressure versus time during flow of gelant solution in the microtube.

The experimental results are shown in Table 7. When the injection rate increased from 0.05 mL·min^−1^ to 0.5 mL·min^−1^, the in situ dynamic gelation time during flow in the microtube decreased from 4.5 h to 3.5 h and the gel breakthrough pressure gradient decreased from 0.04 MPa·m^−1^ to 0.02 MPa·m^−1^.

With the increase of injection rate, the flow gelation time decreases, and the breakthrough pressure gradient of microtube flow gelation decreases. With the increase of injection rate, the gelation system of the gel system is increased by shearing action, the collision probability between the polymer and crosslinker molecules is increased, and the gelation time of the gel is shortened. However, due to the shear effect, the gelation process at the edge shearing edge reduces the strength of gelling, so that the pressure gradient of the flow gel breaks down. When the injection rate is 1.0 mL · min^−1^, the gel has flowed out of the microtube, and the flow gel forming time cannot be determined. Compared with the static gelation in the microtube, due to the existence of shear, the flow gelation time is prolonged and the breakthrough pressure gradient is reduced after gelation.

### 2.4. The Relationship Between Bulk and In Situ Gelation Behavior

According to the bulk gelation behavior and in situ gelation behavior, the relationship between bulk gelation behavior and in situ gelation behavior was compared, as shown in Table 8.

Compared with the in situ static gelation behavior, the in situ dynamic gelation time is prolonged and the breakthrough pressure gradient decreases after gelation. The relationship between the bulk gelation time and in situ gelation time was correlated. The in situ static gelation time in the microtube is 1.2 times that of bulk gelation time in the ampoule bottle, and the in situ dynamic gelation time is nearly 3 times that of ampoule bottles. Therefore, based on the relationship between bulk and in situ gelation behavior, the in situ gelation time in the microtube can be calculated by determining the bulk gelation time according to the ampoule viscosity method, and the reasonable shut in time on site can be estimated according to the static bulk gelation time.

### 2.5. Enhanced Oil Recovery Ability

**Figure 9:**
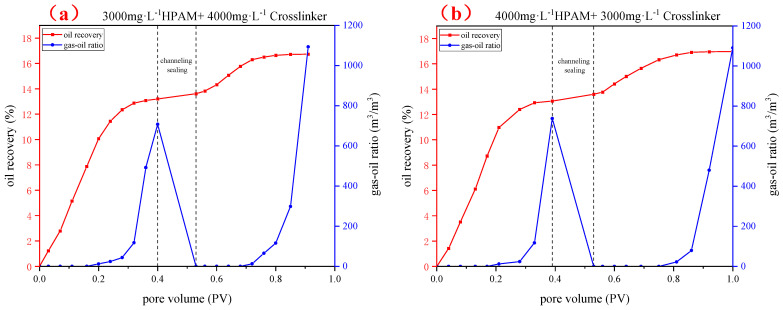

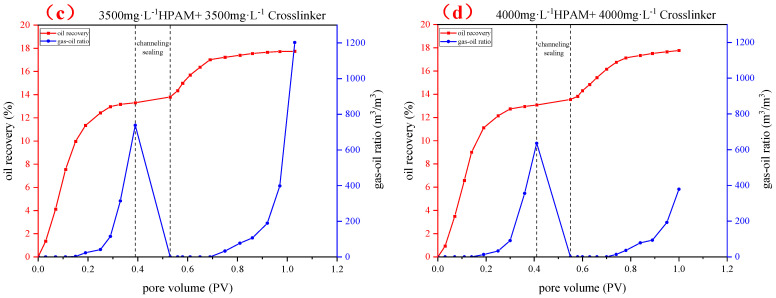
Gas flooding performance curve of an injected gel system with different polymer and crosslinker concentrations.

The change in concentration of the gel system was primarily achieved by altering the gel strength, which in turn affect the plugging efficiency. According to the flooding procedure, the effect of polymer and crosslinker concentrations on the enhanced oil recovery ability of polymer gel used for fractured reservoirs was investigated. In this experiment, with the oil recovery rate increase as the primary indicator, based on the typical gel formula, the injected gel system was fixed at 1.0 FV, and the concentrations of the polymer and crosslinker were adjusted to achieve different levels of gel plugging effects. The experimental results are shown in Figure 9. In this figure, HPAM is an abbreviation for partially hydrolyzed polyacrylamide.

**Figure 10:**
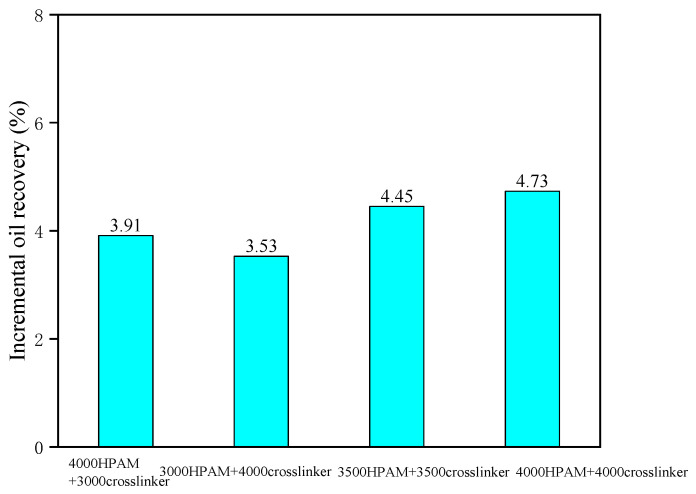
Incremental histogram of oil recovery of different injected gel system concentrations.

The experimental results are shown in Figure 10. From the comprehensive analysis of the flooding performance curves, it can be seen that, during the initial period of continuous CO_2_ injection, the oil recovery continued to increase. When the amount of CO_2_ injected exceeded 0.2 PV, the oil recovery tended to stabilize, and the gas–oil ratio showed a significant improvement. During the injection of the gel system, the gas–oil ratio dropped rapidly, and the oil recovery did not change much. During the subsequent CO_2_ injection period, the oil recovery showed an inflection point of sudden increase, and the gas–oil ratio also increased slightly. When the injected slug volume was 1.0 FV and the concentration ratio of the polymer to the crosslinker was 1:1, as the polymer concentration increased from 3000 mg·L^−1^ to 4000 mg·L^−1^, the incremental oil recovery increased from 3.53% to 4.73%. When the total concentration of polymer and crosslinker was 7000 mg·L^−1^, compared to the other concentrations, the incremental oil recovery of a polymer gel composed of 3500 mg·L^−1^ polymer and 3500 mg·L^−1^ crosslinker was the highest. By analyzing the breakthrough pressure gradient of the gel system with different concentrations, it can be seen that, by changing the ratio of polymer and crosslinker within the appropriate concentration range, the breakthrough pressure changes slightly, ranging from 0.51 to 0.63 MPa/m. Under the same concentration ratio conditions, without a significant increase in breakthrough pressure, the effect of increasing recovery rate is better, indicating that the viscoelasticity of the polymer and crosslinker gel is better under an equal ratio, and the overall plugging effect is better. With the same concentration ratio of polymer and crosslinking agent, when the polymer concentration increases, the gel network structure is more compact, and the strength is higher.

## 3. Conclusions

In this study, the influence of different factors on bulk and in situ gelation process of gelant solution in a microtube were compared. The enhanced oil recovery ability of polymer gel for tight fractured reservoirs was also investigated. The following conclusions were reached:

(1) With the increase of polymer and crosslinker concentration, the bulk gelation time of the gel system was shortened and the gel strength increased. With the increase of temperature from 70 °C to 90 °C, the gelation time of the gel system was greatly shortened, and the gel strength increased.
(2) With the increase of polymer concentration and temperature, the in situ static gelation time and dynamic gelation time of the gel system in the microtube were shortened, and the breakthrough pressure gradient increased after gelation. Compared with the in situ static gelation behavior, the in situ dynamic gelation time was prolonged and the breakthrough pressure gradient decreased after gelation. The relationship between the bulk gelation time and in situ gelation time was correlated. The in situ static gelation time in the microtube was 1.2 times that of the bulk gelation time in the ampoule bottle, and the in situ dynamic gelation time was nearly 3 times that of ampoule bottles.
(3) When the injected slug volume was 1.0 FV and the concentration ratio of the polymer to the crosslinker was 1:1, as the polymer concentration increased from 3000 mg·L^−1^ to 4000 mg·L^−1^, the incremental oil recovery increased from 3.53% to 4.73%.

## 4. Experimental Study

### 4.1. Materials

The polymer used to form the gel was KYPAM-6, provided by Heng Ju Company (Beijing, China). The molecular structure of KYPAM-6 is a high molecular weight polymer, mainly composed of acrylamide, sodium 2-acrylamido-2-methylpropanesulfonate, 1-vinyl-2-pyrrolidone, and non-ionic hydrophobic monomers. This polymer was prepared through a polymerization reaction and has the properties of temperature resistance, salt resistance and hydrolysis resistance. The molecular weight and hydrolysis degree of the polymer are 2600 × 10^4^ and 20%, respectively. The polymer solution was prepared by synthetic formation brine with salinity of total dissolved solids (TDS). The detailed ionic composition of the synthetic formation brine is listed in Table 9.

The organic crosslinker used in this study was phenolic resin that was prepared with phenol and formaldehyde under the catalysis of sodium hydroxide. The reaction equation of the crosslinker preparation is shown in Equation (1). The crosslinking group -CH_2_OH of the phenolic resin crosslinker can react with the amide group -CONH_2_ of the polymer to form a phenolic resin gel with a three-dimensional network structure. The crosslinking equation is shown in Equation (2) [39,40].

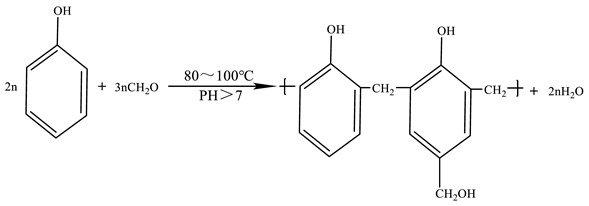
(1)

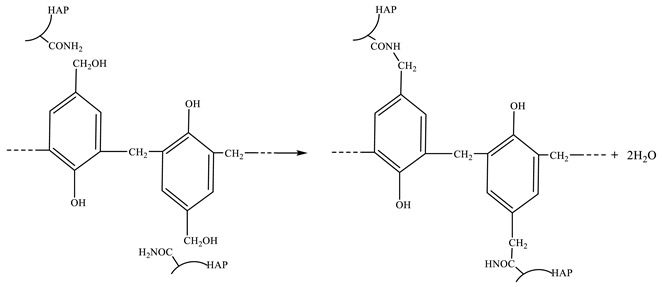
(2)

**Figure 11:**
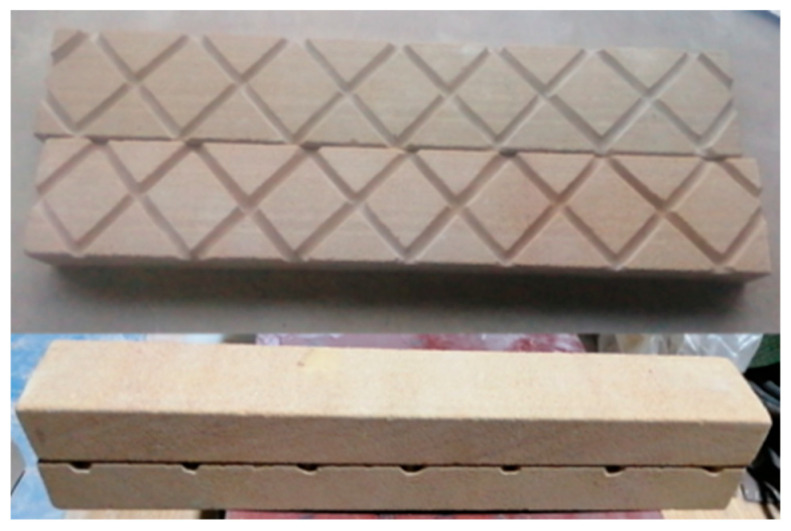
Design and physical image of complex fracture core model.

Organically crosslinked gels tend to be more stable. Long stainless microtubes of different lengths were used to investigate the in situ gelation behavior. A fractured core physical model (4.5 cm × 4.5 cm × 30 cm) was used to investigate the enhanced oil recovery ability of polymer gel used for CO_2_ channeling, as shown in Figure 11. In order to fully simulate the morphology of fractures, this experiment used columnar cores to create fractures by carving fractures on the split wall surface.

### 4.2. Methods

#### 4.2.1. Gelant Solution Preparation

The gelant solution composed of polymer and phenolic resin crosslinker was prepared as follows: (1) Firstly, the polymer solution was prepared by adding polymer powder into the synthetic formation brine under mechanical stirring for about 8 h. (2) Secondly, the gelant solution was obtained by mixing the phenolic resin crosslinker with the polymer solution under mechanical stirring for about 0.5 h. The concentration of polymer and crosslinker ranged from 3000 mg·L^−1^ to 4000 mg·L^−1^_._

#### 4.2.2. Bulk Gelation Behavior Test

The bulk gelation behavior of phenolic gel was studied by the bottle test method, that is, the changes in viscosity of gelant solution during the gelation process were monitored as a function of time. The viscosity of the gelant solution was determined by a Brookfield DV-II viscometer. The Brookfield DV II viscometer can measure viscosity in the range of 1.0~6,000,000 mPa·s, with a rotational speed of 0.01–200 rpm divided into 54 grades. The viscosity of the gelant solution versus time was measured by using different rotors at a rotational speed of 6 rpm. The gelation time is defined as the time corresponding to the inflection point between the induction period and the gelation period when the viscosity of gelant solution increases obviously. The gel strength is characterized by the final viscosity. The bulk gelation behavior tests were conducted at 70 °C and 90 °C.

### 4.3. In Situ Gelation Behavior Test in Microtube

The in situ static and dynamic gelation behavior of phenolic resin gel was studied by conducting long stainless microtube flow experiments.

#### 4.3.1. Experimental Method of In-Situ Static Gelation Behavior

**Figure 12:**
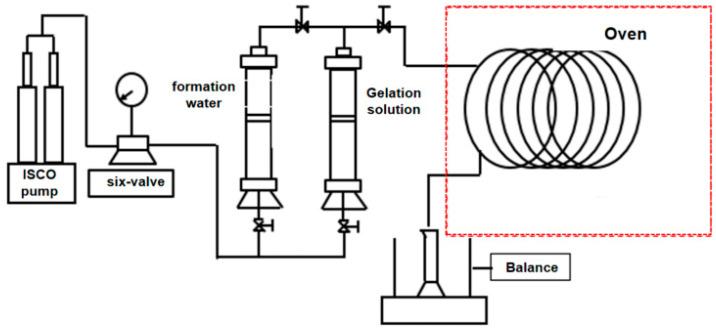
Apparatus of the microtube flow experiment.

In this study, a stainless microtube (Φ 1.0 mm × 20 m) was used to simulate the fractured porous media. The experimental apparatus of the microtube flow is shown in Figure 12. And the procedures of the in situ static gelation behavior test are as follows: (1) 1.0 pore volume (PV) gelant solution was injected into the microtube at a rate of 0.25 mL/min. (2) Then the microtube was put into an oven at different temperatures (70 °C and 90 °C) for different standing times to gelation. Then the microtube was taken out at regular intervals and water flooding was conducted to measure the change of pressure versus time. (3) According to the relationship between the pressure and standing time, the breakthrough pressure and breakthrough pressure gradient under different standing times can be obtained. The breakthrough pressure gradient is calculated according to the breakthrough pressure and microtube length. (4) According to the relationship between breakthrough pressure and breakthrough pressure gradient over time, when the breakthrough pressure and breakthrough pressure gradient of the system begin to rise significantly, the time corresponding to the inflection point between the induction stage and the gelation stage is the microtube in situ static gelation time. Due to the limitation of long gelation time at 70 °C and the length of the microtube model, in order to better analyze and compare the gelation behavior under bulk and in situ gelation conditions, the gelation property of gelant solution composed of 4000 mg·L^−1^ polymer and 4000 mg·L^−1^ crosslinker was studied at 90 °C.

#### 4.3.2. Experimental Method of in-Situ Dynamic Gelation Behavior

In order to simulate the dynamic gelation process of gelant solution in fractured porous media, a physical model of the dynamic gelation process was established as follows: The inner diameter and length of the stainless steel microtube were 1.0 mm and 100 m. Then 1.0 PV gelant solution was continuously injected into the microtube, and the change of injection pressure versus time was recorded during the injection process of gelant solution. The time corresponding to the inflection point of rapid increase in injection pressure was defined as the dynamic gelation time in the microtube. In order to determine the dynamic gelation strength of the gel system in the microtube, after 1.0 PV gelant solution was injected, water flooding was subsequently carried out to determine the breakthrough pressure. The breakthrough pressure was defined as the maximum injection pressure, that is, the strength of the gel in the microtube after dynamic gelation was determined.

### 4.4. Enhanced Oil Recovery Ability Evaluation

**Figure 13:**
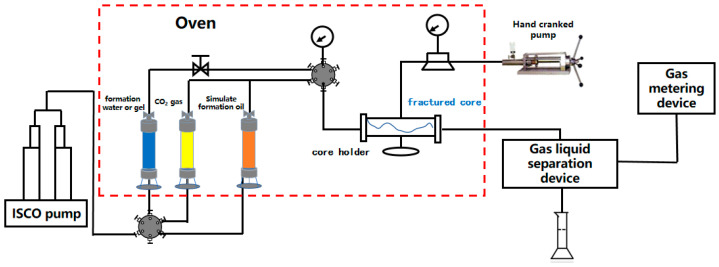
Schematic diagram of core flooding experiment.

The designed fractured core model (as shown in Figure 11) was used to evaluate the enhanced oil recovery ability of polymer gel. The experimental apparatus of core flooding is shown in Figure 13. The core flooding experimental procedures were as follows: (1) The core was dried and weighed. Then the core was vacuumed to saturate the simulated oil. (2) Initial CO_2_ flooding period: Under the back pressure of 10 MPa, CO_2_ gas was injected at a constant rate of 1 mL·min^−1^ and the oil production and the gas–oil ratio were recorded. (3) The gelant solution was injected and the gelation period measured. When the production gas–oil ratio reached 500 m^3^/m^3^, 1.0 FV(fracture volume) a polymer gel system with different concentrations was injected into the fractured core and shut in for 24 h until gelation. (4) Subsequent CO_2_ flooding period: Under the back pressure of 10 MPa, the CO_2_ gas was injected at a constant rate of 1 mL·min^−1^ and the oil production and the gas–oil ratio were recorded again. When the production gas–oil ratio reached 1000 m^3^/m^3^, the experiment was stopped and the incremental oil recovery was calculated.

## Figures and Tables

**Table 1:** The effect of polymer and crosslinker concentration on bulk gelation behavior (70 °C).

No.	Polymer Concentration /mg·L^−1^	Crosslinker Concentration /mg·L^−1^	Polymer/Crosslinker Concentration Ratio	Gelation Time /h	Final Gel Strength/mPa·s
1	3000	3000	1.0	40	11,006
2	3500	3000	1.17	30	22,538
3	4000	3000	1.33	25	25,483
4	3500	3500	1.0	20	26,583
5	3500	4000	0.875	17.5	38,457

**Table 2:** The effect of temperature on bulk gelation time and gel strength.

Temperature /°C	Polymer Concentration /mg·L^−1^	Crosslinker Concentration /mg·L^−1^	Gelation Time /h	Final Gel Strength/mPa·s
70	4000	4000	10.0	41,245
90	4000	4000	1.25	58,553

**Table 3:** The effect of temperature on in situ static gelation time and gel strength of the microtube.

Temperature /°C	Polymer Concentration /mg·L^−1^	Crosslinker Concentration /mg·L^−1^	In Situ Static Gelation Time/h	Breakthrough Pressure Gradient/MPa·m^−1^
70	4000	4000	12.0	0.138
90	4000	4000	1.5	0.365

**Table 4:** The effect of polymer concentration on in situ gelation time and gel strength of the microtube (90 °C).

Temperature /°C	Polymer Concentration /mg·L^−1^	Crosslinker Concentration /mg·L^−1^	In Situ Static Gelation Time/h	Breakthrough Pressure Gradient/MPa·m^−1^
90	3000	4000	3.5	0.21
	3500	4000	2.5	0.265
	4000	4000	1.5	0.365

**Table 5:** Comparison of gelation time of gel systems at different temperatures (70 °C and 90 °C).

Temperature/°C	Polymer Concentration/mg·L^−1^	Crosslinker Concentration/mg·L^−1^	In Situ Dynamic Gelation Time/h	In Situ Dynamic Gel Strength/MPa·m^−1^
70	4000	4000	32.5	0.0325
90	4000	4000	4.5	0.04

**Table 6:** The in situ dynamic gelation time and strength of gelant solution under different injection rate (90 °C).

Temperature /°C	Injection Rate /mL·min^−1^	Polymer Concentration /mg·L^−1^	Crosslinker Concentration/ mg·L^−1^	In Situ Dynamic Gelation Time/h	In Situ Dynamic Gel Strength /MPa·m^−1^
90	0.25	3000	4000	5.8	0.0132
		3500	4000	4.7	0.0158
		4000	4000	3.9	0.026

**Table 7:** The in situ dynamic gelation time and strength of gelant solution under different injection rates (90 °C).

Temperature /°C	Polymer Concentration /mg·L^−1^	Crosslinker Concentration /mg·L^−1^	Injection Rate /mL·min^−1^	In Situ Dynamic Gelation Time/h	Breakthrough Pressure Gradient /MPa·m^−1^
90	4000	4000	0.05	4.5	0.04
			0.25	3.9	0.026
			0.5	3.5	0.02

**Table 8:** Relationship between bulk and in situ gelation time of the gel system at different temperatures.

Temperature/°C	Injection Rate /mL·min^−1^	Polymer Concentration/mg·L^−1^	Crosslinker Concentration/mg·L^−1^	Gelation Time/h		
				Bulk Gelation	In Situ Static Gelation	In Situ Dynamic Gelation
70	0.05	4000	4000	10	12	32.5
90	0.05	4000	4000	1.25	1.5	4.5

**Table 9:** The ionic composition of the synthetic brine.

Ionic Type	HCO^3−^	Ca^2+^	Mg^2+^	Cl^−^	Na^+^	K^+^	TDS/mg·L^−1^
Ionic concentration	156.1	2347.0	204.5	19,077.3	9320.3	51.4	31,254.6

## Data Availability

The data presented in this study are openly available in article.

## References

[1] Zou C., Yang Z., Zhu R., Zhang G., Hou L., Wu S., Tao S., Yuan X., Dong D., Wang Y. (2015). Progress in China’s unconventional oil & gas exploration and development and theoretical technologies. Acta Geol. Sin.-Engl. Ed..

[2] Sydansk R.D., Seright R.S. (2007). When and where relative permeability modification water-shutoff treatments can be successfully applied. SPE Prod. Oper..

[3] Al-Obaidi S.H., Khalaf F. (2020). Development of traditional water flooding to increase oil recovery. Int. J. Sci. Technol. Res..

[4] Kharrat R., Alalim N., Ott H. (2023). Assessing the Influence of Fracture Networks on Gas-Based Enhanced Oil Recovery Methods. Energies.

[5] Ganguly S. (2010). Displacement of Cr (III)–partially hydrolyzed polyacrylamide gelling solution in a fracture in porous media. Transp. Porous Media.

[6] McCool S., Li X., Wilhite G.P. Effect of shear on flow properties during placement and on syneresis after placement of a polyacrylamide-chromium acetate gelant. Proceedings of the SPE International Conference on Oilfield Chemistry.

[7] Medeiros R.S., Biswas D., Suryanarayana P. Impact of thief zone identification and shut-off on water production in the Nimr field. Proceedings of the SPE/IADC Managed Pressure Drilling and Underbalanced Operations Conference and Exhibition.

[8] Botermans C.W., Dalrymple E.D., Dahl J., Smith D. Chemical Systems for Water and Gas Control: Terminology, Evaluation Methods, Candidate Selection, and Expectations. Proceedings of the SPE International Conference on Oilfield Chemistry.

[9] Sydansk R.D. (1990). A newly developed chromium (lll) gel technology. SPE Reserv. Eng..

[10] Bryant S.L., Bartosek M., Lockhart T.P. (1997). Laboratory evaluation of phenol—Formaldehyde/polymer gelants for high-temperature applications. J. Pet. Sci. Eng..

[11] Chauveteau G., Tabary R., Renard M., Omari A. Controlling in-situ gelation of polyacrylamides by zirconium for water shutoff. Proceedings of the SPE International Conference on Oilfield Chemistry.

[12] Chung T., Bae W., Nguyen N., Dang C., Lee W., Jung B. (2011). A review of polymer conformance treatment: A successful guideline for water control in mature fields. Energy Sources Part A Recovery Util. Environ. Eff..

[13] El-Karsani K.S., Al-Muntasheri G.A., Hussein I.A. (2014). Polymer systems for water shutoff and profile modification: A review over the last decade. SPE J..

[14] Li L., Zhou X., Su Y., Xiao P., Chen Z., Zheng J. (2022). Influence of heterogeneity and fracture conductivity on supercritical CO_2_ miscible flooding enhancing oil recovery and gas channeling in tight oil reservoirs. Energy Fuels.

[15] Zhang J., Zhang H.X., Ma L.Y., Liu Y., Zhang L. (2020). Performance evaluation and mechanism with different CO_2_ flooding modes in tight oil reservoir with fractures. J. Pet. Sci. Eng..

[16] Hao H., Hou J., Zhao F., Song Z., Hou L., Wang Z. (2016). Gas channeling control during CO_2_ immiscible flooding in 3D radial flow model with complex fractures and heterogeneity. J. Pet. Sci. Eng..

[17] Zhao F., Hao H., Hou J., Hou L., Song Z. (2015). CO_2_ mobility control and sweep efficiency improvement using starch gel or ethylenediamine in ultra-low permeability oil layers with different types of heterogeneity. J. Pet. Sci. Eng..

[18] Liu Y., Bai B., Wang Y. (2010). Applied technologies and prospects of conformance control treatments in China. Oil Gas Sci. Technol.–Rev. d’IFP Energ. Nouv..

[19] Mokhtari M., Ozbayoglu E.M. Laboratory investigation on gelation behavior of xanthan crosslinked with borate intended to combat lost circulation. Proceedings of the SPE International Production and Operations Conference and Exhibition.

[20] Shen H., Yang Z., Li X., Peng Y., Dong Z. (2021). Co2-responsive agent for restraining gas channeling during co2 flooding in low permeability reservoirs. Fuel.

[21] Deng J.-N., Zhao H., Zheng H., Zhuang Y., Wei K., Yuan H., Deng Z., Gao Y., Zhou X., Yu T. (2025). CO_2_-responsive preformed particle gels with high strength for CO_2_ conformance control in heterogeneous reservoirs. Fuel.

[22] Lemaire P., Alenzi A., Lee J., Beckman E., Enick R. (2021). Thickening CO_2_ with direct thickeners, CO_2_-in-Oil emulsions, or nanoparticle dispersions: Literature review and experimental validation. Energy Fuels.

[23] Lockhart T.P. (1994). Chemical properties of chromium/polyacrylamide gels. SPE Adv. Technol. Ser..

[24] Solbakken J.S., Aarra M.G. (2021). CO_2_ mobility control improvement using N2-foam at high pressure and high temperature conditions. Int. J. Greenh. Gas Control.

[25] Sengupta B., Sharma V., Udayabhanu G. (2012). Gelation studies of an organically cross-linked polyacrylamide water shut-off gel system at different temperatures and pH. J. Pet. Sci. Eng..

[26] Prasad S.K., Sangwai J.S., Byun H.-S. (2023). A review of the supercritical CO_2_ fluid applications for improved oil and gas production and associated carbon storage. J. CO_2_ Util..

[27] Wang T., Wang L., Qin H., Zhao C., Bai Z., Meng X. (2022). Identification of Gas Channeling and Construction of a Gel-Enhanced Foam Plugging System for Oxygen-Reduced Air Flooding in the Changqing Oilfield. Gels.

[28] Bai Y., Pu W., Jin X., Shen C., Ren H. (2024). Review of the micro and Macro mechanisms of gel-based plugging agents for enhancing oil recovery of unconventional water flooding oil reservoirs. J. Mol. Liq..

[29] He H., Wang Y., Zhang J., Xu X., Zhu Y., Bai S. (2015). Comparison of gelation behavior and morphology of resorcinol–hexamethylenetetramine–HPAM gel in bulk and porous media. Transp. Porous Media.

[30] Esfahlan M.S., Khodapanah E., Tabatabaei-Nezhad S.A. (2021). Comprehensive review on the research and field application of preformed particle gel conformance control technology. J. Pet. Sci. Eng..

[31] Yu H.Y., Liu X.J., Ji W.J., Gao H.T., Xu B.B. (2014). Effect of Dynamic Gelation of Polymer Gel in Porous Media: Permeability. Appl. Mech. Mater..

[32] Jousset F., Green D., Willhite G., McCool C. Effect of high shear rate on in-situ gelation of a xanthan/Cr (III) system. Proceedings of the SPE Improved Oil Recovery Conference.

[33] Gommes C.J., Roberts A.P. (2008). Structure development of resorcinol-formaldehyde gels: Microphase separation or colloid aggregation. Phys. Rev. E—Stat. Nonlinear Soft Matter Phys..

[34] Yadav U.S., Mahto V. (2013). Investigating the effect of several parameters on the gelation behavior of partially hydrolyzed polyacrylamide–hexamine–hydroquinone gels. Ind. Eng. Chem. Res..

[35] Haiyang Y., Yefei W., Jian Z., Peng L., Shenglong S. (2015). Dynamic gelation of HPAM/Cr (III) under shear in an agitator and porous media. Oil Gas Sci. Technol.–Rev. d’IFP Energ. Nouv..

[36] Yu H., Ma Z., Tang L., Li Y., Shao X., Tian Y., Qian J., Fu J., Li D., Wang L. (2022). The effect of shear rate on dynamic gelation of phenol formaldehyde resin gel in porous media. Gels.

[37] Xin Y., Li B., Li Z., Li Z., Wang B., Wang X., Zhang M., Li W. (2025). Gas channeling control with CO_2_-responsive gel system in fractured low-permeability reservoirs: Enhancing oil recovery during CO_2_ flooding. Sep. Purif. Technol..

[38] Wang H., Yang C., Zhang Y., Wang C. (2024). Preparation and Effect of CO_2_ Response Gel for Plugging Low-Permeability Reservoirs. Gels.

[39] You Q., Zhou W., Wang Y., Zhao F., Ji C., Fan S. (2010). The reaction process and influencing factors of hydrophobic associative polymer/phenolic resin gel. Oilfield Chem..

[40] He H., Wang Y., Zhang J. (2015). Novel gel with controllable strength for in-depth conformance control: Bulk gelation performance and propagation properties in porous media. J. Dispers. Sci. Technol..

